# Relationship between the Gut Microbiome and Osteoarthritis Pain: Review of the Literature

**DOI:** 10.3390/nu13030716

**Published:** 2021-02-24

**Authors:** Eleuterio A. Sánchez Romero, Erika Meléndez Oliva, José Luis Alonso Pérez, Sebastián Martín Pérez, Silvia Turroni, Lorenzo Marchese, Jorge Hugo Villafañe

**Affiliations:** 1 Musculoskeletal Pain and Motor Control Research Group, Faculty of Health Sciences, Universidad Europea de Madrid, 28670 Madrid, Spain; erikamelendezoliva@gmail.com (E.M.O.); joseluis.alonso@universidadeuropea.es (J.L.A.P.); sebastian.martin@universidadeuropea.es (S.M.P.); 2 Universidad Europea de Madrid, Faculty of Biomedical and Health Sciences, Department of Physiotherapy, 28670 Madrid, Spain; 3 Musculoskeletal Pain and Motor Control Research Group, Faculty of Health Sciences, Universidad Europea de Canarias, C/Inocencio García 1 38300 La Orotava, 38300 Tenerife, Canary Islands, Spain; 4 University of Bologna, Department of Pharmacy and Biotechnology, Via Belmeloro 6, 40126 Bologna, Italy; silvia.turroni@unibo.it; 5 IRCCS Fondazione Don Carlo Gnocchi, Piazzale Morandi 6, 20141 Milan, Italy; lmarchese@dongnocchi.it

**Keywords:** osteoarthritis, gastrointestinal microbiome, dysbiosis

## Abstract

**Background**: Osteoarthritis (OA) is the most common form of chronic pain in Europe (34%), representing a great economic and social cost to society. There are studies that suggest an intestine–brain–articulation axis and hint at the existence of low-grade intestinal inflammation in OA, which would be related to an alteration of the microbiota and to the impairment of the epithelial barrier, with leakage of the microbial components. Purpose: The purpose of this study was to review the association between gut microbiome and pain in the OA population through a review of the literature. Methods: A literature search was conducted to identify all available studies on the association between the gut microbiome and pain in the OA population, with no publication date limit until September 2020 and no language limit, in the MEDLINE, CINAHL, Web of Science and Cochrane Central Register of Controlled Trials databases. Results: Only three of 2084 studies detected and analyzed by performing the proposed searches in the detailed databases, were finally selected for this review, of which one was with and two were without intervention. These studies only weakly support a relationship between the gut microbiome and OA, specifically a correlation between certain taxa or microbial products and the inflammatory landscape and severity of OA symptoms, including knee pain. Conclusions: Despite encouraging results, this review highlights the paucity of high-quality studies addressing the potential role of the gut microbiome in OA-related pain, along with the disparity of the techniques used so far, making it impossible to draw firm conclusions on the topic.

## 1. Introduction

Osteoarthritis (OA) is the most common form of chronic pain in Europe (34%), representing a great economic and social cost to society [1]. Likewise, there is a directly proportional relationship of suffering greater pain intensity with medical comorbidities, obesity, lower educational level, depression, belonging to a younger generation and joint degeneration [2]. However, in recent years, OA has been more closely related to central sensitization (CS) mechanisms [3,4,5]. This sensitization in spinal cord and brain shows in experimental studies how patients with OA are more sensitive to experimental noxious stimuli at body sites away from their affected joints compared to patients without OA pain [6]. One possible mechanism that relates to CS is widespread inflammation [7]. On the other hand, the severity of the joint disease is not related to the pain experienced [8]. Clinically, pain along with joint stiffness are the most disabling symptoms experienced by OA patients [9]. Often these patients go through episodes of recurrent pain or exacerbation.

It is well-known that our lifestyle greatly influences the prevalence of OA. Unhealthy diets with low fiber content, high fat and sugar content along with sedentary lifestyles make OA more prevalent today. Several studies link poor nutritional diets to the occurrence of low-grade inflammation in the intestinal mucosa [10]. The same risk factors are also well-known to contribute to altering the gut microbiota towards potentially dysbiotic configurations associated with the disease [11].

The human microbiome, i.e., the set of microbial ecosystems populating the different niches of the organism, is a well-established factor that communicates with the different systems and organs of the human body, influencing our physiology [12]. In particular, the alteration, i.e., dysbiosis, of the gut microbiota, if persistent over time, can promote excessive porosity of the epithelial barrier and thus the leakage of microorganisms and their products into the circulatory system [13]. There are studies that explain the existence of low-grade intestinal inflammation in OA and suggest a potential role for the microbiome in OA-related pain [14,15]. The elevation of the systemic levels of lipopolysaccharide (LPS) may explain a possible correlation between the gut microbiome and OA [16]. In addition, stress and pain could be directly responsible for modulating the microbiota, through the release of hormones and sympathetic neurotransmitters that alter gut physiology, microbial gene expression and signaling [17], thus contributing to the increase in intestinal permeability. Although the mechanisms of action remain to be defined, the presence of inflammatory products and microbial DNA in the joint would show a direct relationship between the intestine and inflammatory arthropathies [18,19]. While experts stress the importance of harnessing this microbiome-related information to design new and more effective therapeutic strategies for OA [20], little and sparse evidence is currently available in the literature.

In an attempt to clarify the state of the art on this topic, here we carried out a review of the literature to search for a possible association between the gut microbiome and pain in the OA population.

## 2. Materials and Methods

This is a literature review of studies investigating or reporting an association between gut microbiome and pain in the OA population. PRISMA [21] guidelines were followed during the design, search and reporting stages of this review.

### 2.1. Search Strategy

A literature search was conducted to identify all available studies on the association between the gut microbiome and pain in the OA population, with no publication date limit until September 2020 and no language limit, in the MEDLINE, CINAHL Web of Science and Cochrane Central Register of Controlled Trials databases. In MEDLINE, the search string was (“gastrointestinal microbiome” [MeSH Terms] OR (“gastrointestinal” [All Fields] OR “dysbiosis” [All Fields]) AND (“pain” [MeSH Terms] OR “pain” [All Fields]) OR “gut microbiome” [All Fields]) OR (“dysbiosis” [MeSH Terms] AND (“osteoarthritis” [MeSH Terms] OR “osteoarthritis” [All Fields]) OR “gut microbiome” [All Fields]) OR (“dysbiosis” [MeSH Terms] OR “dysbiosis” [All Fields]) AND (“arthralgia” [MeSH Terms] OR “arthralgia” [All Fields] OR (“joint” [All Fields] AND “pain” [All Fields]) OR “joint pain” [All Fields]). Similar research equations were used to consult CINAHL, Web of Science and Cochrane Central Register of Controlled Trials databases. Additional records were searched through other sources to complement the database findings; for example, manual searches of reference lists of relevant literature reviews and indexes of peer-reviewed journals were performed.

Two independent researchers (E.A.S.R. and E.M.O.) conducted the searches and evaluated all the articles found by title and abstracts, and subsequently the full-text publications to determine their eligibility. This procedure was performed by each researcher involved in this part of the study (E.A.S.R. and E.M.O.) according to the inclusion and exclusion criteria of the research, and a third author (J.H.V.) resolved discrepancies. The list of references in each article was screened in order to find any additional original articles.

### 2.2. Study Selection

#### 2.2.1. Type of Studies

The types of studies included randomized controlled trials in patients with OA pain who received assessment or intervention on their gut microbiome, and observational studies (cohort studies, case–control studies and case series) with human patients, without restrictions regarding the date of publication. We excluded from analysis all repeated articles, case reports, letters to editor, pilot studies, editorials, technical notes and review articles. Articles written in any language were included.

#### 2.2.2. Type of Participants

The participants in selected studies had to be adults (18 years of age or older) with a diagnosis of symptomatic OA (OA pain).

#### 2.2.3. Data Extraction

Data extraction was performed independently by two authors (E.A.S.R. and E.M.O.), and in case of disagreement, a third author (J.H.V.) was in charge of resolving discrepancies. Reviewers were not blind to the reporting of the results of each published article, the name of the authors, institutions or the scientific journal. A standardized work template was used to extract and detail all the information related to the methodology of each study, the sample size and mean age of the study patients, sex distribution, year and country of publication, recruitment, the association of pain related to OA, the different follow-ups over time, the clinical outcome measures and the detailed main results. The Cochrane Handbook for Systematic Reviews of Interventions-Version 5.1.0 [22] was used to develop these sections. This form was pilot-tested for reliability using a representative sample of the studies to be reviewed.

#### 2.2.4. Quality Assessment

The PEDro scale and the Cochrane Risk of Bias Tool were used to assess the methodological quality of the clinical trials finally included for scoring in the present review.

To assess the methodological quality and risk of bias of the nonrandomized studies, we used the methodological index for nonrandomized studies (MINORS) [23]. This scoring system includes eight items for nonrandomized studies and four additional items for comparative studies. Each item is scored between 0 and 2, and the maximum attainable score is 16 and 24 for nonrandomized studies and comparative studies, respectively. Two authors independently answered the questions with 0 (not reported), 1 (reported but inadequate) or 2 (reported and adequate); any disagreement of the authors (E.A.S.R. or E.M.O.) was resolved by discussion, and in case of conflicting scores, the third reviewer (J.H.V.) resolved to make the decision.

#### 2.2.5. Study Selection

**Figure 1:**
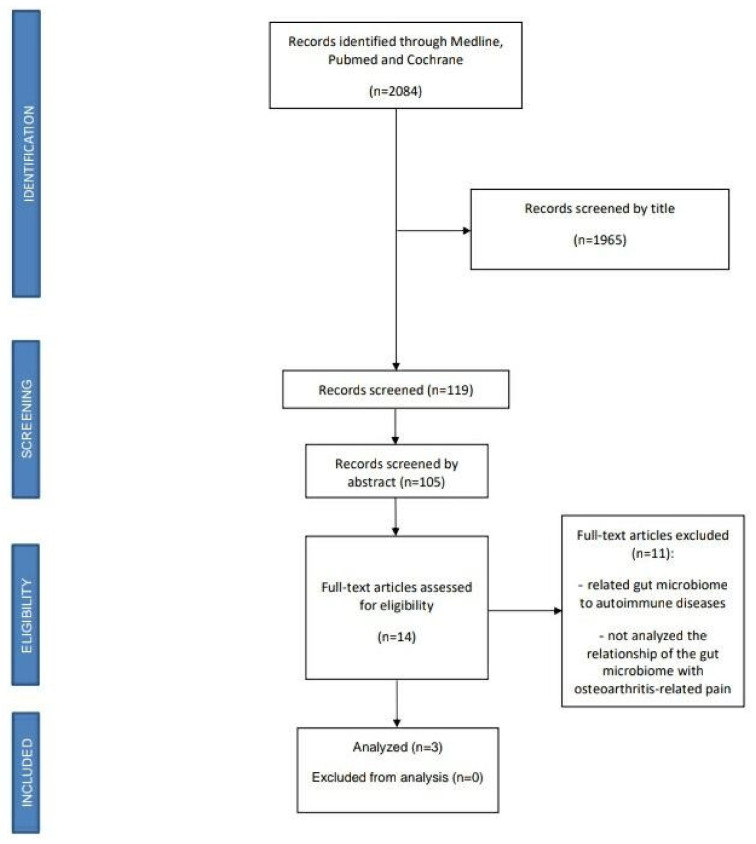
PRISMA flow diagram.

A total of 2084 studies were detected and analyzed by performing the proposed searches in the detailed databases. After eliminating duplicates and analyzing the titles and abstracts of the remaining articles, 14 full-text articles were evaluated for possible inclusion in the present study. Ultimately, 11 of these manuscripts were excluded for not analyzing the relationship of the gut microbiome with OA-related pain. Thus, three studies were finally selected for this review, Figure 1.

## 3. Results

The three included studies were conducted in Australia [24], the Netherlands [25], the United States and China [26], and published from 2013 to 2019.

### 3.1. Study Characteristics

The characteristics of the included studies are presented in Table 1. The type of studies included 1 clinical trial [24], 1 case–control study [25] and 1 cohort study [26].

### 3.2. Risk of Bias within Studies

The PEDro Scale and the Cochrane Risk of Bias Tool for assessing risk of bias in randomized trials were used to score the clinical trial [24], obtaining a score of 7 out of 11 and 2, respectively (Table 2). Besides, the MINORS Scale was used to score the case–control study and the cohort study, which yielded mean scores of 18 out of 24 and 14 out of 16, respectively. Overall, the three studies, clinical trial [24], case–control [25] and cohort [26], were of good quality (Table 3).

### 3.3. Data from Studies

#### 3.3.1. Association between Microbiome and OA-Related Pain in Articles with Intervention

Coulson et al. (2013) [24] analyzed the effect of 3000 mg/day of green-lipped mussel extract (GLM) or 3000 mg/day of glucosamine sulphate (GS) for 12 weeks on the composition of the gut microbiome, through viable plate counting and MALDI-TOF mass spectrometry-based colony identification. In addition, the changes produced in the arthritic scores in the Western Ontario McMaster Universities Arthritis Index (WOMAC), Lequesne Algofunctional Indices, Quality of Life (SF12) and Gastrointestinal Symptom Rating Scale (GSRS) were assessed at baseline, 6 and 12 weeks (T0, T6 and T12) in patients with knee OA. The results of this study showed that both groups (GLM and GS) improved significantly (*p* < 0.05) in all WOMAC measures (pain, stiffness, flexibility and function) and GSRS score after 12 weeks of intervention. Although the changes in the gut microbiome were not significant, there was a notable decrease in both groups in Clostridia sp., which were reported to trigger T cell-driven gut inflammation and arthritis in mouse models [27,28]. This decrease was consistent with reduced inflammation and collaterally with improvement in OA and gastrointestinal symptoms.

#### 3.3.2. Association between Microbiome and OA-Related Pain in Articles without Intervention

Boer et al. (2019) [25] investigated the relationship between joint pain and fecal microbiome composition, and OA-related knee pain in the Rotterdam Study, a large population-based cohort study. Stool samples were collected from 1427 patients and the microbiome was analyzed through 16S rRNA gene-based Illumina sequencing. The authors noted that microbiome ß-diversity was significantly associated with knee WOMAC scores. In particular, a greater relative abundance of *Streptococcus* was observed in individuals with higher pain values on the WOMAC scale, regardless of tobacco and/or alcohol consumption and BMI. This association was robust (*p* = 1.4 × 10^−4^) and driven by local inflammation in the knee joint.

Huang et al. (2016) [26], in a cohort study with 25 patients, evaluated the association of LPS, a proinflammatory component of Gram-negative bacteria, with the severity of inflammation, symptoms and radiographic abnormalities in knee OA. In their study, they demonstrated the presence of LPS in synovial fluid of the knee and serum, which had a significant correlation with the abundance of activated macrophages in the knee, the severity of radiographic OA and joint symptoms.

## 4. Discussion

To date, the association between the gut microbiome and OA-related pain has been sought in three high-quality studies, of which one was with [24] and two were without intervention [25,26]. Such studies only weakly support a relationship, specifically a correlation between the levels of certain taxa or microbial products, namely LPS, and the inflammatory landscape and severity of OA symptoms, including knee WOMAC pain. The major microbial taxa hypothesized to be involved include *Clostridium* and *Streptococcus* species, with the former having been shown to promote Th17 cells and drive arthritis [28] and the latter postulated to lead to increased knee pain through activation of local or systemic macrophages [25]. These assumptions are consistent with the previous study by Huang et al. [26], which associates the presence of LPS and LPS-binding protein in both the serum and synovial fluid of OA patients with that of activated macrophages in the knee, OA severity and joint symptoms, mainly pain. It is therefore reasonable to speculate that a dysbiotic gut microbiome contributes to eliciting a local and systemic inflammatory state, also through the leakage of microbial products or metabolites across an impaired epithelial barrier, ultimately inducing or exacerbating OA-related pain.

However, as regards microbiome profiling techniques, it should be noted that in the clinical trial [24], the authors used a traditional culture-dependent approach, resulting in the identification of only a limited number of aerobes and anaerobes, which are generally subdominant in the healthy adult gut microbiome and therefore have little potential of biological relevance (i.e., coliforms, yeasts, *Enterococcus*, *Streptococcus*, *Staphylococcus*, *Bacteroides*, *Prevotella*, *Eubacterium*, *Lactobacillus*, *Bifidobacterium*, *Clostridium*). This may partly explain why the authors struggle to find significant differences. On the other hand, the case-control study [25] is a recent study in a large population-based cohort, employing cutting-edge technologies, whose main findings (i.e., the association between the relative abundance of *Streptococcus* and knee WOMAC pain and inflammation) were replicated in an independent cohort. Finally, the cohort study [26] only examined LPS levels, thus providing no information on the possible proinflammatory imbalances of the gut microbiome.

## 5. Conclusions

In conclusion, this review highlights the paucity of high-quality studies addressing the potential role of the gut microbiome in OA-related pain, along with the disparity of the techniques used so far, making it impossible to draw firm conclusions on the topic. Despite these limitations and the obvious associative nature of the studies, all the available data consistently hint at the establishment of a dysbiotic, proinflammatory microbiome profile in OA patients, which may play a role in the severity of symptoms, particularly pain, sustaining inflammatory sequelae both locally and systemically. Further studies in larger cohorts, using the most advanced next-generation sequencing technologies for microbiome profiling, will be needed to confirm these encouraging data and provide insights into the mechanisms underlying the relationship between gut microbiome and OA-related pain. Such mechanisms should possibly be validated in an animal model. In the near future, the targeted manipulation of the gut microbiome can be expected to become an integral part of current pain-relieving strategies, thereby providing benefits to the OA population.

## Figures and Tables

**Table 1:** Characteristics of included studies.

Author, Year	Aim of the Study	Study Design	ParticiPants	Treatment	Outcome Measures	Reported Results
Coulson et al. (2013) [24]	Evaluate how the efficacy of nutraceuticals in treating OA may be altered according to the different microbiota profiles of the gastrointestinal tract and allow the formulation of a hypothesis that partly explains the inconsistent and controversial results of osteoarthritis (OA) clinical studies with green-lipped mussel (GLM) and glucosamine.	Clinical trial	40 patients (29 women) Mean age: 58.6 ± 8.9 years Inclusion criteria: Patients with knee OA eligible for ACR.	3000 mg/day green-lipped mussel extract (GLM) or 3000 mg/day glucosamine sulphate for 12 weeks.	- Microbiota analysis in feces (T_0_ and T_12_) through viable plate counting and MALDI-TOF mass spectrometry-based colony identification. - WOMAC, Lequesne algofunctional index, SF12 score (quality of life) measure and GSRS (T_0_, T_6_ and T_12_) - Other: BP, height, BMI, WHI and CRP.	Results Significant improvement (*p* < 0.05) in all WOMAC [pain, stiffness, flexibility and function] and GSRS measures. Although without significant changes in the microbiota, in both groups ↓ *Clostridium* and *Staphylococcus* and ↑ *Lactobacillus*, *Streptococcus* and *Eubacterium*. In the GLM group ↑ *Bifidobacterium* and ↓ *Enterococcus* and yeasts. In the GS group ↓ *Bacteroides* and ↑ yeasts and coliforms, most notably *Escherichia coli*.
Boer et al. (2019) [25]	Verify the relationship between joint pain and the composition of the gastrointestinal microbiome, and knee pain related to osteoarthritis in the Rotterdam Study.	Case-Control	1427 patients (821 women) Mean age: 56.8 ± 5.9 years Inclusion criteria: Patients with knee OA (cases) and without knee OA (controls) from Rotterdam Study.	-	-- 16S rRNA gene-based Illumina sequencing for microbiome profiling. - WOMAC Index.	Results - Microbiome ß-diversity was significantly associated with knee WOMAC scores. - A greater relative abundance of *Streptococcus* was found in individuals with higher pain values on the WOMAC scale, regardless of tobacco, alcohol consumption and BMI. - There was a significant association between the relative abundance of *Streptococcus* spp. and knee WOMAC-pain scores (*p* = 1.4 × 10^−4^). This association was robust and driven by local inflammation in the knee joint.
Huang et al. (2016) [26]	To analyze the relationship of lipopolysaccharide (LPS), a decisive proinflammatory product of the microbiome, with the level of inflammation, symptoms and radiographic alterations in osteoarthritis of the knee.	Cohort study	25 patients from the Etarfolatide cohort (18 women) Mean age: 62.4.1 ± 15.8 years Inclusion criteria: radiographic knee OA (unilateral or bilateral [K/L] grade 1–4).	-	- LPS was measured using the EndoZyme Assay (recombinant factor C based), carefully optimized for systemic and synovial fluid analyses. - LBP was tested in both serum and synovial fluid for association with OA phenotypic outcomes (commercial sandwich ELISA kit). - Models were adjusted for age, gender and BMI. - WOMAC Index.	Results - Serum LPS and LBP were associated with the abundance of activated macrophages in the knee joint capsule (*p* = 0.01) and synovium (*p* = 0.036). - SF LPS and LBP were associated with the abundance of activated macrophages in the synovium (*p* = 0.001 and *p* = 0.021, respectively). - Serum LPS, LBP and SF LPS were associated with knee osteophyte severity (*p* = 0.030, *p* = 0.017 and *p* = 0.001, respectively). - SF LPS was positively associated with knee joint space narrowing severity (*p* < 0.001) and total WOMAC score (*p* = 0.008). - Serum LBP tended to show a positive association with knee pain score (*p* = 0.076). - SF LBP was significantly associated with self-reported knee pain score (*p* = 0.039). - Both LPS and LBP concentrations were significantly lower in SF than in paired serum (*p* < 0.0001). - Serum LPS and LBP concentrations were highly correlated (*p* < 0.001) and individually correlated with BMI (*p* < 0.017) and plasma sCD14 (*p* < 0.001).

ACR: American College of Rheumatology; BMI: body mass index; BP: blood pressure; GLM: Green-lipped mussel extract; GS: glucosamine sulphate; GSRS: Gastrointestinal Symptom Rating Scale; K/L: Kellgreen/Lawrence grading system; LBP: LPS binding protein; LPS: lipopolysaccharide; OA: Osteoarthritis; SF: Synovial fluid; SF-12: 12-Item Short Form Survey; T_0_: At the beginning; T_6_: At 6 weeks; T_12_: At 12 weeks; WHI: Waist hip index; WOMAC: Western Ontario and McMaster Universities Osteoarthritis Index; ↓ Decrease; ↑ Increase.

**Table 2:** Methodological quality evaluation of the clinical trials using the PEDro scale and the Cochrane risk of bias tool for assessing the risk of bias in randomized trials.

Bias AnalysisTtable for RCTs (Cochrane Collaboration)												
AUTHORS	Selection bias		Realization bias		Detection bias		Wear bias		Notification bias		Others	OUTCOME
Coulson et al. (2012) [24]	Yes		Yes		No		No		No		No	2
**Scale “Physiotherapy Evidence Database (PEDro)” to analyze the methodological quality of clinical studies**												
AUTHORS	Specified selection criteria	Randomization	Hidden assignment	Similar groups to start	Blinded patients	Blinded therapists	Blinded raters	Outcomes 85%	Treatment or intention to treat	Comparison between groups	Point measures variability	OUTCOME
Coulson et al. (2012) [24]	Yes	Yes	No	Yes	No	No	No	Yes	Yes	Yes	Yes	7

**Table 3:** Methodological index for nonrandomized studies (MINORS) to assess the methodological quality and risk of bias of the observational included studies. The items are scored 0 (not reported), 1 (reported but inadequate) or 2 (reported and adequate). The global ideal score being 16 for noncomparative studies and 24 for comparative studies.

Methodological Index for Nonrandomized Studies (MINORS)													
AUTHORS	Clearly stated aim	Inclusion of consecutive patients	Prospective collection of data	Endpoints appropriate to the aim of the study	Unbiased assessment of the study endpoint	Follow-up period appropriate to the aim of the study	Loss to follow up less than 5%	Prospective calculation of the study size	Adequate control group	Contemporary groups	Baseline equivalence of groups	Adequate statistical analyses	OUTCOME
Boer et al. (2019) [25]	2	2	2	2	0	0	2	2	1	2	1	2	18
Huang et al. (2016) [26]	2	2	2	2	0	0	2	2	0	0	0	2	14

## Data Availability

The data presented in this study are available on request from the corresponding authors.
